# High Physiological Concentrations of Progesterone Reverse Estradiol-Mediated Changes in Differentiation and Functions of Bone Marrow Derived Dendritic Cells

**DOI:** 10.1371/journal.pone.0153304

**Published:** 2016-04-11

**Authors:** Fangming Xiu, Varun C. Anipindi, Philip V. Nguyen, Jeanette Boudreau, Hong Liang, Yonghong Wan, Denis P. Snider, Charu Kaushic

**Affiliations:** McMaster Immunology Research Center, Department of Pathology and Molecular Medicine, McMaster University, Michael G. DeGroote Center for Learning and Discovery, Hamilton, Ontario, Canada L8N 3Z5; Oklahoma Medical Research Foundation, UNITED STATES

## Abstract

Female sex steroids, estradiol (E2) and progesterone (P4), play a key role in regulating immune responses in women, including dendritic cell (DC) development, and functions. Although the two hormones co-occur in the body of women throughout the reproductive years, no studies have explored their complex combinatorial effects on DCs, given their ability to regulate each other’s actions. We examined murine bone marrow derived dendritic cells (BMDC) differentiation and functions, in the presence of a wide range of physiological concentrations of each hormone, as well as the combination of the two hormones. E2 (10^−12^ to 10^-8^M) enhanced the differentiation of CD11b^+^CD11c^+^ DCs from BM precursor cells, and promoted the expression of CD40 and MHC Class-II, in a dose-dependent manner. In contrast, P4 (10^−9^ to 10^-5^M) inhibited DC differentiation, but only at the highest concentrations. These effects on BMDCs were observed both in the presence or absence of LPS. When both hormones were combined, higher concentrations of P4, at levels seen in pregnancy (10^-6^M) reversed the E2 effects, regardless of the concentration of E2, especially in the absence of LPS. Functionally, antigen uptake was decreased and pro-inflammatory cytokines, IL-12, IL-1 and IL-6 production by CD11b^+^CD11c^+^ DCs, was increased in the presence of E2 and these effects were reversed by high concentrations of P4. Our results demonstrate the distinct effects of E2 and P4 on differentiation and functions of bone marrow myeloid DCs. The dominating effect of higher physiological concentrations of P4 provides insight into how DC functions could be modulated during pregnancy.

## Introduction

Dendritic cells (DCs) play a central role in both innate and acquired immune responses [1] [2]. These cells are derived from hematopoietic stem cells and differentiate into myeloid and lymphoid-type lineages. Most peripheral tissues including mucosal epithelium, are seeded with myeloid lineage DCs, that express specific differentiation markers, dependent on the tissue type [3] [4]. The most common markers of the myeloid lineage DC are CD11c, CD11b, and CD103 [4]. Under normal homeostatic conditions, tissue DCs have a short lifespan, and are constantly replaced by fresh DC replenished from BM precursors. Under non-inflammatory conditions, tissue DCs are relatively immature in their ability to initiate adaptive immune responses. Because of their location at the internal and external body surface, and their ability to endocytose and process antigens from invading pathogens, the tissue DCs play a critical role during innate responses, as first responders to infection, and subsequently, following activation and migration to tissue-draining lymph nodes in directing and coordinating T cell responses. It therefore follows, that altered physiologic conditions, such as hormonal changes, stress, or injury can likely alter both the differentiation of DCs and their immune functions.

Sex hormones, estrogen (E2) and progesterone (P4) are known to alter immune function, including response to infection and autoimmune pathogenesis [5] [6] [7] [8,9]. Our own work has demonstrated that the quality of immune response to HSV-2 infection in mice is distinct based on the hormonal priming at time of immunization [8,9] [10]. This implied that both E2 and P4 influenced the type of immune responses initiated. We therefore decided to examine of the effects of E2 and P4 on dendritic cell differentiation and functions from BM precursors.

Work by others has looked separately at E2 and P4 for effects on DC development and function [7] [11]. Kovats and co-workers have demonstrated that E2 can preferentially direct differentiation of precursor cells into myeloid DCs, characterized by CD11c expression and moderate expression of CD11b, and then further promotes their differentiation to functional DC, in vitro [12] [13]. The functionally mature DCs promoted by E2, expressed higher levels of MHC II, CD40, and cytokines IL-12 and IL-6, and presented antigen to naïve CD4 T cells [12]. Others have focused on P4 effects on DC differentiation and immune function. P4 altered the cytokine profile of mature DC, typically inhibiting IL-6, IL-12 and TNF-α production [14] [7]. Other studies have indicated that progesterone increased in vitro differentiation of mouse DC from BM precursors [15], but that it inhibited in vitro maturation of DC, reducing MHC II and IL-12 expression [16]. Mature DCs from spleen of female mice have reduced cytokine secretion and co-stimulator expression during the progesterone-high time of the hormonal cycle [17]. Thus, opposing effects of E2 and P4 on DC maturation and function have been observed when the hormones are examined individually. However, no studies have examined effects of combining both hormones in physiologic ranges, given that these hormones are present in varying ratios at different times of the reproductive cycle as well as during pregnancy. Because the systemic levels of E2 and P4 are continuously modified in the female reproductive cycle, both the functions of DCs in active immune processes and the development of DC subsets from precursors, would be cyclically affected with changes in levels and combination of both E2 and P4. Conditions such as pregnancy with prolonged high progesterone levels may promote a prolonged state of altered DC function. Therefore, in this study we examined effect of each hormone separately and in combination and found that at high physiological concentrations, equivalent to those seen in pregnancy, P4 can reverse E2 effects on both differentiation and functions of DCs.

## Material and Methods

### Cell culture medium and reagents

All the reagents except those specified, were purchased from Sigma-Aldrich (Oakville, Ontario, Canada). DCs were cultures in RPMI 1640 (Invitrogen, Burlington, ON, Canada) supplemented with 10% FBS (Hyclone, ThermoFisher Scientific, USA), 2mM glutamine, 100U penicillin/0.1mg streptomycin/ml, 10mM HEPES buffer, 50μM 2-ME and 1mM sodium pyruvate. Hormone-deficient medium was RPMI 1640 lacking phenol red (Invitrogen, Burlington, ON) and 10% of charcoal-dextran-treated FBS (Invitrogen, Burlington, ON) instead of regular FCS. Stock solution of water soluble E2 (10^-3^M) and P4 (10^-2^M) were prepared in PBS and stored at -80°C, thawed and diluted into BM culture at varying concentrations when needed. Stock solutions of ICI 182,780 (1mM) Sigma-Aldrich, Oakville, Ontario, Canada, RU-486 (0.1μM) (Sigma-Aldrich, Oakville, Ontario, Canada or AL082D06 (D06) (1mM) (Caledon Laboratory Chemicals, Georgetown, Ontario, Canada) were prepared in DMSO and diluted into BM cultures at varying concentrations in the presence of exogenous E2 and P4 respectively. Collagenase A, DNase I, FITC-Dextran (MW 40K) and LPS from Escherichia coli were purchased from Sigma-Aldrich. Recombinant murine GM-CSF was purchased from PeproTech (Rocky Hill, NJ, US) and stock solution of 400 μg/ml was stored at -80°C.

### Animals and ovariectomy

Female C57BL/6 mice were purchased from Charles River Laboratories (St Constant, Quebec, Canada). All animals were housed maximum five per cage and maintained on a constant light: dark 12:12 cycle. 6–8 wk-old mice were bilaterally ovariectomized (OVX) under Ketamine (80mg/kg body mass)-xylazine (8mg/kg body mass) injectable anesthesia. Mice were allowed to recover for two weeks prior to being used for further experimentation. Estradiol receptor knockout (ERKO) C57BL/6 mice were kindly provided by Dr. Pierre Chambon (Université Louis Pasteur, France). ERKO mice were also ovariectomized, 2 weeks before use.

### Ethics Statement

Animals were euthanized by CO_2_ inhalation, as per approved protocols. All animals in this study were housed at the McMaster Central Animal facility, and the protocols used were approved by the McMaster University Animal Research Ethics Board (AREB) as per AUP # 14-09-40 in accordance with Canadian Council of Animal Care (CCAC) guidelines.

### Generation of DC from BM cells

BM cells were generated as described with slight modification[18]. BM cells were isolated from the femurs and tibiae of ovariectomized C57BL/6 mice and cultured in hormone-deficient RPMI 1640 with 40ng/ml of mouse GM-CSF. Total BM cells were seeded at 3x10^5^ cells per ml in 2ml of media in a 24-well plate. On day 3, half of the culture media were removed and replaced with fresh hormone-deficient media containing 40ng/ml of GM-CSF. To activate DC, at day 6, LPS (5ng/ml, final concentration) was added into the culture for further 24 hr. BM cells were also isolated from ER knockout ovariectomized C57BL/6 mice and cultured as mentioned above.

### Monoclonal antibodies and flow cytometry

All antibodies for flow cytometry for DC markers and intracellular staining were obtained from BD Bioscience (Mississauga, Canada). They included APC-conjugated rat anti-mouse CD11a, PE-CY7-conjugated hamster anti-mouse CD11c, PE-conjugated rat anti-mouse CD40, FITC-conjugated rat anti-mouse I-A^b^, APC-conjugated rat anti-mouse IL-12, IL-6 and TNF-α, and their respective isotype control antibodies. For DCs staining, cells were pre-incubated with Fc receptor blocker for 10 min at room temperature (RT), and then stained with antibodies against CD11b, CD11c, CD40 and MHCII in PBS containing 0.2% BSA. Labeled cells were run on a FACSCanto or LSRII flow cytometer and data were analyzed by FlowJo (v. 7.15) software.

### E2 and P4 treatment of DC and blocking with antagonists

Varying doses of E2 (10^-12^M to 10^-8^M) or P4 (10^−9^ M to 10^-5^M) were added into first day of BMDC culture in a 24 well-plate, containing BMDCs at concentration of 3X10^5^ cells/ml. To test if P4 inhibited E2’s effects, varying doses of P4 were added to the BMDC culture containing either 10^-9^M or 10^-11^M of E2 on the first day of culture. E2 and P4 were replenished at day 3 when media was changed. For sex hormone blocking experiments, varying doses of ICI 182 780 (1nM to 100nM) or RU-486 (100nM to 10μM) or AL082D06 (D06) (1uM to 100uM) were added at the beginning of the culture with 1nM of E2 or 1μM of P4 respectively. Addition of DMSO alone (0.15%, final concentration) to the BM culture was used as the control. At day 3, E2 and P4 and the antagonists were also replenished when media was changed.

### Antigen uptake assay

Antigen uptake assay using FITC-Dextran as model antigen was performed as described [19]. On Day 5 of BMDC culture, FITC-Dextran (Sigma) was added to the BM culture at the final concentration of 200 μg/ml for 2 h at 37°C. Cells added with the same concentration of FITC-Dextran were pulsed at 0°C as the background control. Uptake was stopped by adding ice-cold FACS buffer followed by two washes. The cells from each group were collected and incubated for 10 min at room temperature with FcR blocker to block unspecific binding. Then the cells were stained with APC-conjugated anti-CD11b antibody, PE-CY7-conjugated anti-CD11c antibody. Internalization ability was evaluated at the percentage of FITC-positive cells gated on the CD11b^+^CD11c^+^DCs.

### Measurement of cytokine production by multi-analyte cytokine assay

At day 6 or day 7, BM culture supernatants treated with varying doses of E2 or P4 or their combinations either with LPS or without LPS treatment were collected. The concentrations of IL-10, IL-1β, IL-6, TNFα, IL-12, IL-8 and IFNγ in these supernatants were determined by multiplex cytokine assay kit from Meso Scale Discovery (Gaithersburg, Maryland, US). TGF-β in these supernatants was determined by mouse TGF-β Quantikine ELISA kit from R&D systems (Minneapolis, MN, US). The concentrations (pg/ml, mean±SD) were normalized to the percentage of CD11b^+^ CD11c^+^ DCs in each treatment.

### Intracellular staining of BMDC

Intracellular staining (ICS) of TNFα, IL-6 and IL-12p70 was performed according to the direction of ICS kit (BD Bioscience). BM cells were treated either with E2 (10^-11^M or 10^-9^M) or P4 (10^-8^M or 10^-6^M) or E2 plus P4 (10^-11^M E2 +10^-6^M P4 or 10^-9^M E2 + 10^-6^M P4). At day 6, LPS was added to the culture (final concentration, 5ng/ml) for further 24 hours. In the last 6 hours of LPS activation, 1 μl of GolgiPlug (BD Bioscience) (a protein transport inhibitor) was added into the culture for each well. Cells were harvested from each well. After Fc receptor blocking, cells were stained with antibodies for CD11c and CD11b. To do intracellular staining, pelleted cells were first resuspended with 250 μl Fixation/Permeabilization solution for 20 min at 4°C. Then, fixed and permeabilized cells were stained with antibodies for IL-6, IL-12p70 and TNF-α. 200,000 events per sample were collected with a BD LSRII and analyzed by FlowJo software.

### Statistics

All comparisons were performed by one way ANOVA analysis using GraphPad Prism version 4 (GraphPad Software, San Diego CA), with a P value ≤0.05 considered as statistically significant.

## Results

### Contrasting effects of E2 and P4 on the differentiation of CD11b^+^ CD11c^+^ APCs in BM cell cultures

In order to exclude any influence of endogenous or exogenous hormones, we modified a previously described ex vivo culture system for GM-CSF-mediated generation of DC from bone marrow [20]. Bone marrow cells from ovariectomized mice were isolated and grown in medium containing charcoal-stripped serum that is devoid of lipophilic materials, including steroid hormones, which could directly affect the cultures. This approach excluded undefined effects of both endogenous and exogenous sex hormones.

**Fig 1:**
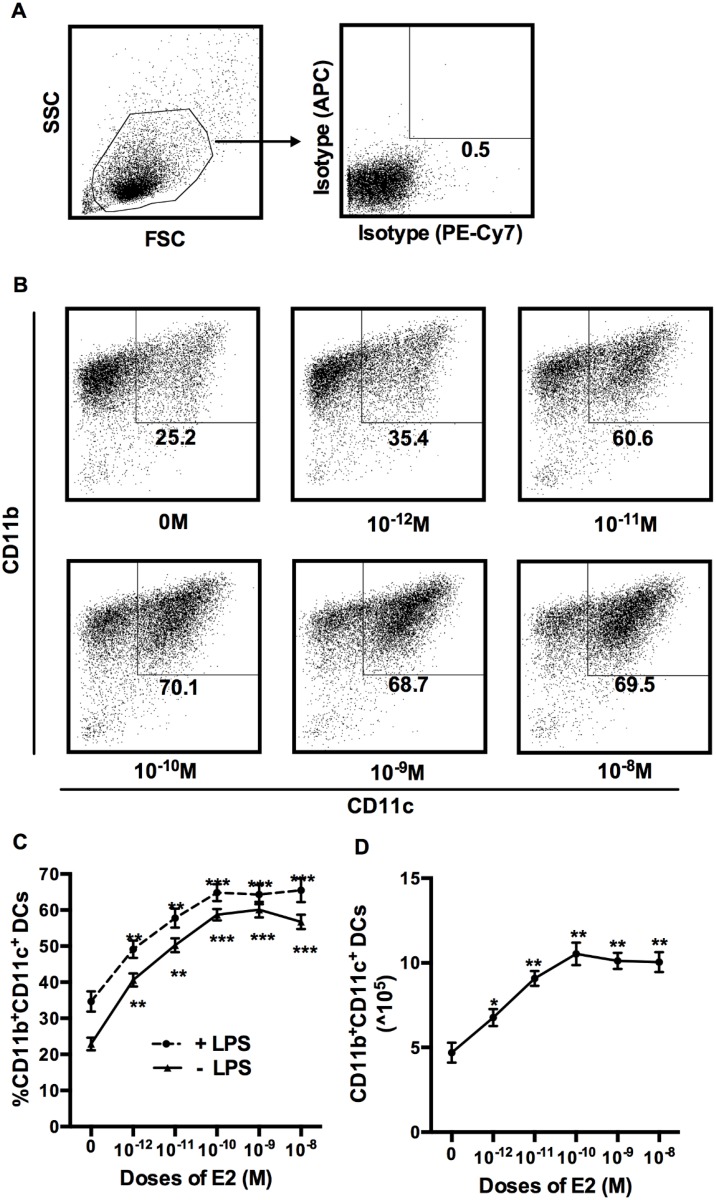
Effects of E2 on the differentiation of DC from BM. BM cell isolated from OVX mice were cultured in sex hormone-deficient medium with murine GM-CSF alone, or in the presence of varying concentrations of E2 (A, B, C, D). Initial gating for mononuclear cells is shown in (A). Data in (B, C, D) are from cells collected at end of culture, and stained with Abs for CD11b and CD11c. Representative dot plot data are shown in (B). Data for percentages of CD11b^+^CD11c^+^ DCs from cultures with different concentrations of E2 and treated with (dashed) or without (solid) LPS are shown in (C). Absolute numbers of CD11b^+^ CD11c^+^ DCs cultured with different concentrations of E2 are shown in (D). Data shown is Mean ± SD of 6 independent experiments. Statistical significance is indicated by * p<0.05; **p<0.01; *** p<0.001 compared with untreated control.

Unfractionated BM cells were isolated from ovariectomized C57BL/6 female mice and cultured in steroid hormone-deficient media, containing 40ng/ml of GM-CSF, in the absence or the presence of 10^-12^M to 10^-8^M of E2. We used a wide range of E2 concentrations, based on known physiological concentrations in serum and those used in other studies. Serum concentrations of E2 vary between 10^−11^ and 10^−10^ M (20–60pg/ml) during mouse estrus cycle and can be as high as 0.5X 10^−9^ M during pregnancy [21] [22]. In humans, serum E2 levels during pregnancy peak around 1X10^-9^ M (350pg/ml) [23]. Many experimental studies test E2 concentrations in the 10^−9^ to 10^−8^ M range [21] [24] [25]. Therefore we examined the effect of entire range of E2 concentrations on BMDC. At day 6, all cells in culture were collected and stained with antibodies against CD11b and CD11c. As shown in Fig 1B, the percentage of double positive DC’s (CD11b^+^CD11c^+^) at the end of culture was approximately 25% of viable cells, in the absence of any sex hormones. However, addition of as little as 10^-12^M E2, increased the percentage of differentiated DC’s (CD11c^+^). This increase was dose-dependent, in the range of 10^-12^M to 10^-10^M, as doses above 10^-10^M did not further increase the fraction of DC’s (Fig 1B). The effect of E2 on DC differentiation was statistically significant over the whole range of concentrations tested (p<0.01 or <0.001) (Fig 1C and 1D). The addition of LPS to the culture on Day 6, increased the percentage of CD11b^+^CD11c^+^ DC at all the E2 concentrations tested by approximately 10% (Fig 1C). The effect of LPS appeared additive to the effect of E2. To exclude the possibility that the change in percentages was due to alterations in total number of viable cells, we calculated the number of CD11b^+^CD11c^+^ DCs at day 6 of the BMDC culture, using viable cell counts from those cultures. Data from this study indicated that total viable cell numbers varied little (variability range of <10%) over all E2 treatment groups (data not shown). In concurrence with the effects seen on the proportion of BMDCs, the effects of E2 on absolute DC numbers were significant as well (p<0.05 or 0.01), at all concentrations (Fig 1D).

**Fig 2:**
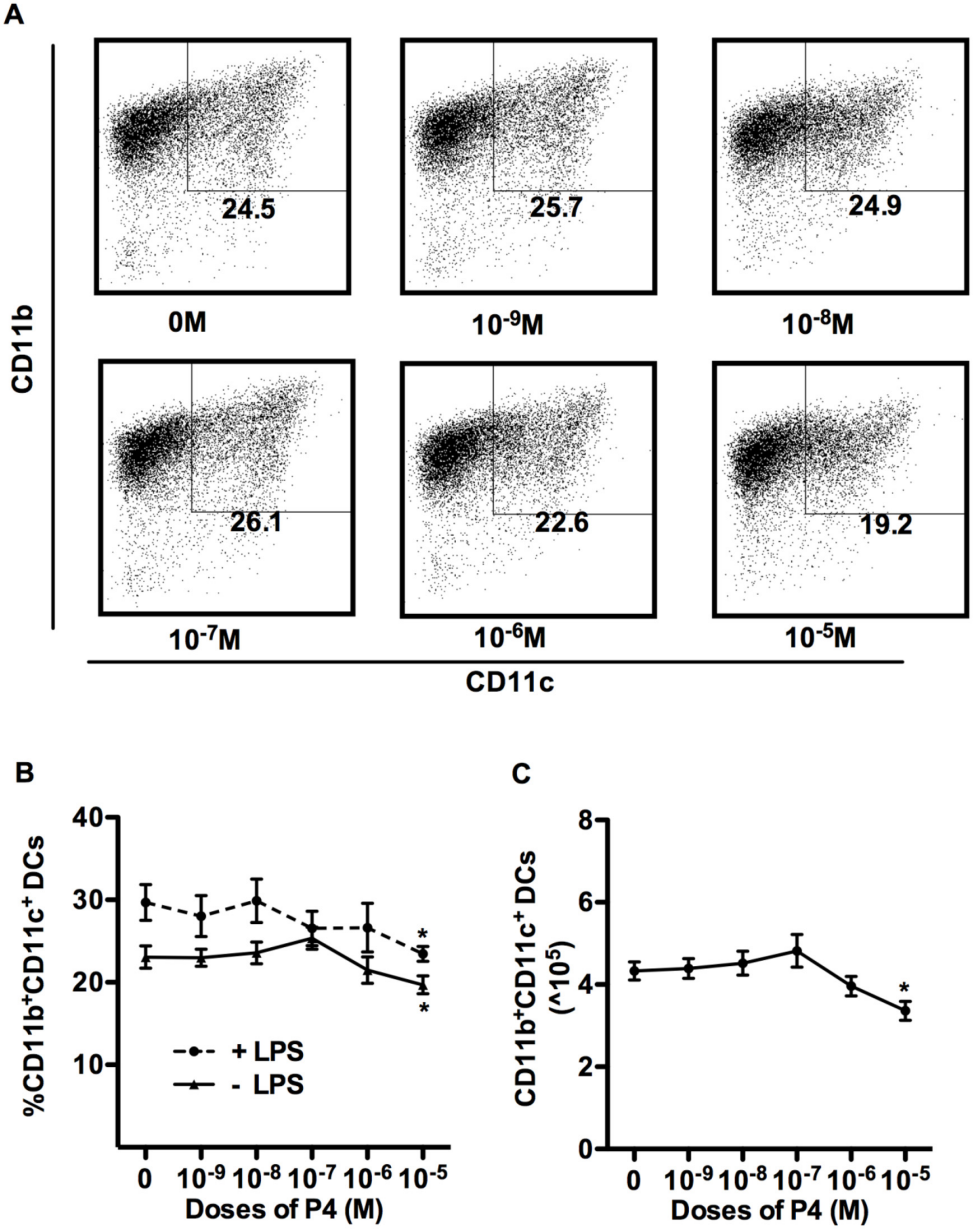
Effects of P4 on the differentiation of APCs from BM. BM cell isolated from OVX mice were cultured in sex hormone-deficient medium with murine GM-CSF alone, or in the presence of varying concentrations of P4. Data in (A, B, C) are from cells collected at end of culture, and stained with Abs for CD11b and CD11c. Initial gating for monocytes as shown in Fig 1A. Representative dot plot data are shown in (A). Percentages of CD11b^+^CD11c^+^ APCs from cultures grown in presence of different concentrations of P4, treated with (dashed) or without (solid) LPS are shown in (B). Absolute numbers of CD11b^+^ CD11c^+^ APCs cultured with P4 are shown in (C). Data show Mean ± SD of 6 independent experiments. Statistical significance is indicated by * p<0.05; **p<0.01; *** p<0.001 compared with untreated control.

Next, we determined the effects of P4 on differentiation of CD11b^+^CD11c^+^ APCs from BM. We used a wide dose range of P4, from 10^-9^M to 10^-5^M. The serum concentration of P4 during the reproductive cycle is between 10^−9^ to 10^−8^ M (2–20ng/ml), with highest levels seen in pregnancy, close to 10^−6^ M (300–400ng/ml) [23] [22] [21]. Levels of progesterone in reproductive tract tissues such as ovary and placenta can be 2–5 fold higher than serum levels [26,27]. Many studies use P4 levels of 10^−6^ to 10^−5^ M for their experiments [16] [24]. Therefore we examined the effects of P4 over this entire dose range. Our results show that for majority of the physiological concentrations tested (10^−9^ to10^-7^M), P4 had no effect on CD11b^+^CD11c^+^ APC differentiation. Only the highest doses of P4 (10^-6^M and 10^-5^M) could inhibit the percentage of differentiated APCs in culture and the decrease was only statistically significant at the highest concentration (Fig 2A and 2B). The decrease in differentiated APCs at highest concentration of P4 was also evident in cultures treated with LPS, where overall percentages of CD11b^+^CD11c^+^ APCs were increased, compared to cultures not treated with LPS (Fig 2B). Only the highest dose of P4 (10^-5^M) was seen to inhibit the APC numbers at the end of culture (Fig 2C).

These initial data indicate that E2 and P4 have different effects on the differentiation of DC. Wide range of physiological concentrations of E2 (10^−11^ to 10^-9^M) promoted CD11b^+^CD11c^+^DCs differentiation from bone marrow. However, P4 for most part had very little effect on DC differentiation at physiological concentrations (10^−9^ to 10^−6^ M) and only the supraphysiological concentrations of P4 (10^-5^M) significantly inhibited CD11b^+^CD11c^+^ APC differentiation from bone marrow. Comparison of dose response data for percentages versus numbers of DC (Figs 1C, 1D and 2B, 2C), indicates close correlation between percentage increase or decrease and total cell numbers across the dose range of E2 and P4. This clearly showed that percentages of cells reflected accurately the actual numbers of DC in various cultures. Therefore, for convenience, data from subsequent experiments was analyzed and presented primarily as differences in the frequency of DC generated in E2 or P4 treated BM cells.

### Contrasting Effects of E2 and P4 on BMDC differentiation in absence or presence of LPS

**Fig 3:**
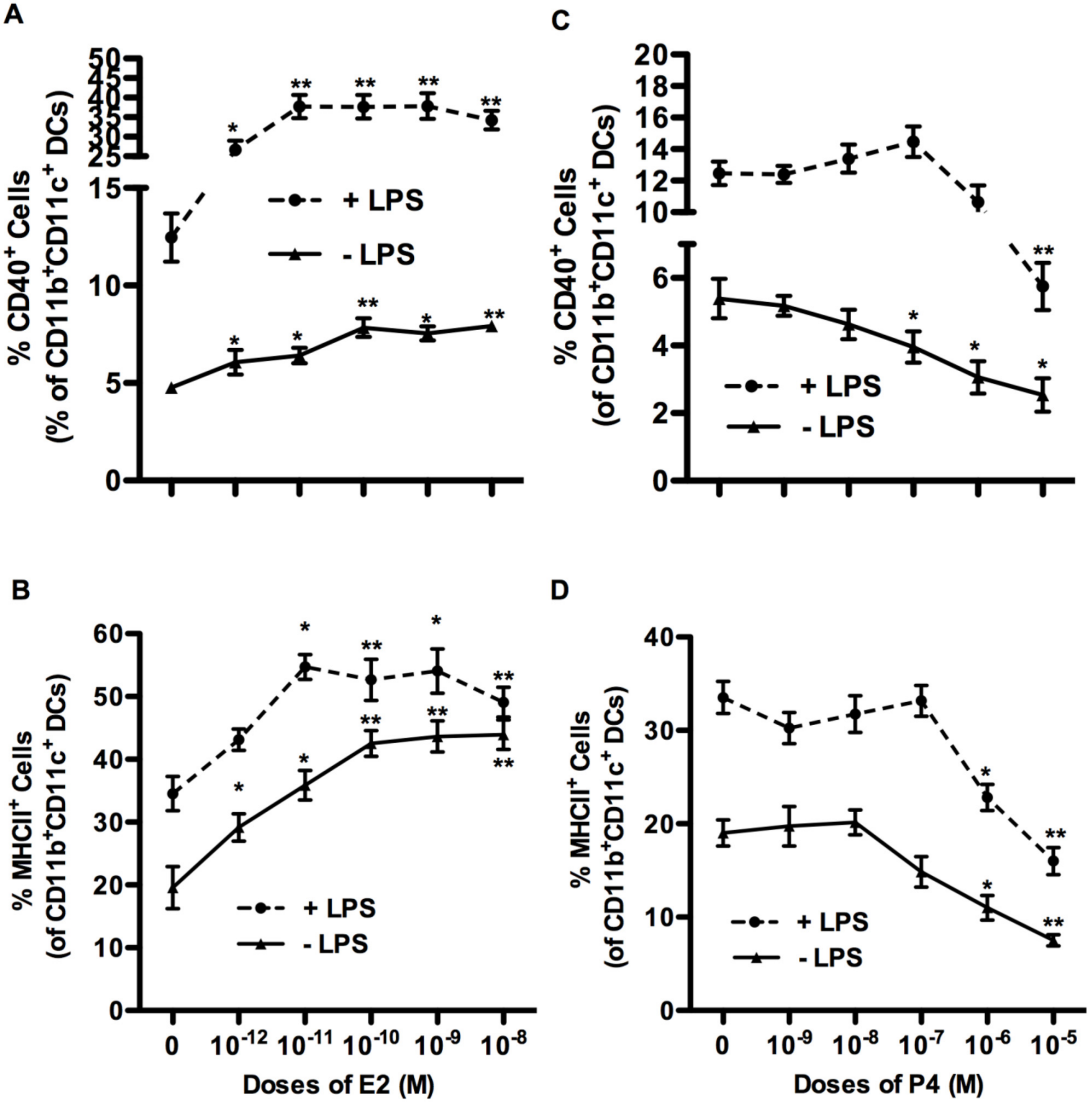
Effects of E2 and P4 on BM APC maturation. BM cell isolated from OVX mice were cultured in sex hormone-deficient medium with murine GM-CSF alone, or in the presence of varying concentrations of E2 (A, B) or P4 (C, D). At end of culture, cells were collected and stained with Abs for CD11b, CD11c, MHC II and CD40 antibodies. Cell culture were treated with (dashed) or without (solid) LPS for last 24 hrs. Y axis represents the percentage of CD40+ (A, C) or MHCII+ (B, D) CD11b^+^CD11c^+^ DCs. Data shown are Mean± SD of 6 independent experiments. Statistical significance is indicated by * p<0.05; **p<0.01 compared with untreated control.

During the process of differentiation into mature DC, DCs undergo a number of phenotypic changes, including increased expression of CD40, CD80, CD86, ICAM-1 and MHC Class-II. To investigate the effects of E2 and P4 on DC differentiation, we chose CD40 and MHCII as the representative markers. On its own, E2 enhanced the frequency of DC expressing CD40, by a factor of 2, from approximately 4% to 8% (Fig 3A). However, the combination of LPS with E2, resulted in a high frequency (30–40%) of CD40 expressing DC, in an E2 dose-dependent manner. Similar to CD40, the percentage of MHCII expressing DCs was increased in the presence of E2, although the direct effects of E2 alone on frequency of MHCII^+^ cells were more prominent, increasing from baseline of 20% (no hormone) to peak levels of 40% at E2 concentration of 10^-10^M (Fig 3B). In the presence of both E2 and LPS, MHCII was expressed on more than 50% of DC. Concentrations at or above 10^-9^M E2 did not further enhance the frequency of CD40 or MHCII expressing DCs.

In contrast to E2, low doses of P4 (10^−9^ and 10^-8^M) had no effect, whereas higher concentrations of P4 inhibited the percentage of CD11b^+^CD11c^+^ APCs expressing CD40 and MHCII (Fig 3C and 3D). The small fractions of MHCII and CD40 expressing cells (20% and 5% respectively) that differentiated in the BMDC cultures grown in the hormone-deficient media were further reduced by a factor of 2–3 in the presence of high dose of P4 (10^−7^ to 10^−5^ M). A similar dose-dependent inhibitory effect of P4 was seen with BM cells stimulated with LPS, although the overall percentage of CD40 and MHCII positive APCs was increased in the presence LPS compared to cultures not treated with LPS. The effects of P4 were statistically significant only at the highest doses, 10^−6^ M and 10^−5^ M.

Taken together, E2 and P4 had contrasting effects on DC differentiation. The whole range of doses of E2 increased the frequency of of CD40 and MHCII expressing CD11b^+^CD11c^+^ DCs. On the contrary, only high doses of P4, corresponding to pregnancy levels (10^-6^M) or supraphysiological (10^-5^M) decreased the frequency of CD11b^+^CD11c^+^ APCs. Given these effects, in subsequent experiments, we chose to examine the effect of the whole dose range of P4 (10^−9^ to 10^-5^M), in combination with either a low dose of E2 (10^-11^M) corresponding to physiological levels seen during normal reproductive cycle or high dose of E2 (10^-9^M) corresponding to levels during pregnancy[21]. In some experiments, we combined the two hormones either at the pregnancy concentrations (10^-9^M E2 with 10^-6^M P4) or those seen during normal reproductive cycle (10^-11^M E2 with 10^-8^M P4).

### Pregnancy levels of P4 can reverse the effects of E2 on the frequency of differentiated BMDC, in the presence or absence of LPS

**Fig 4:**
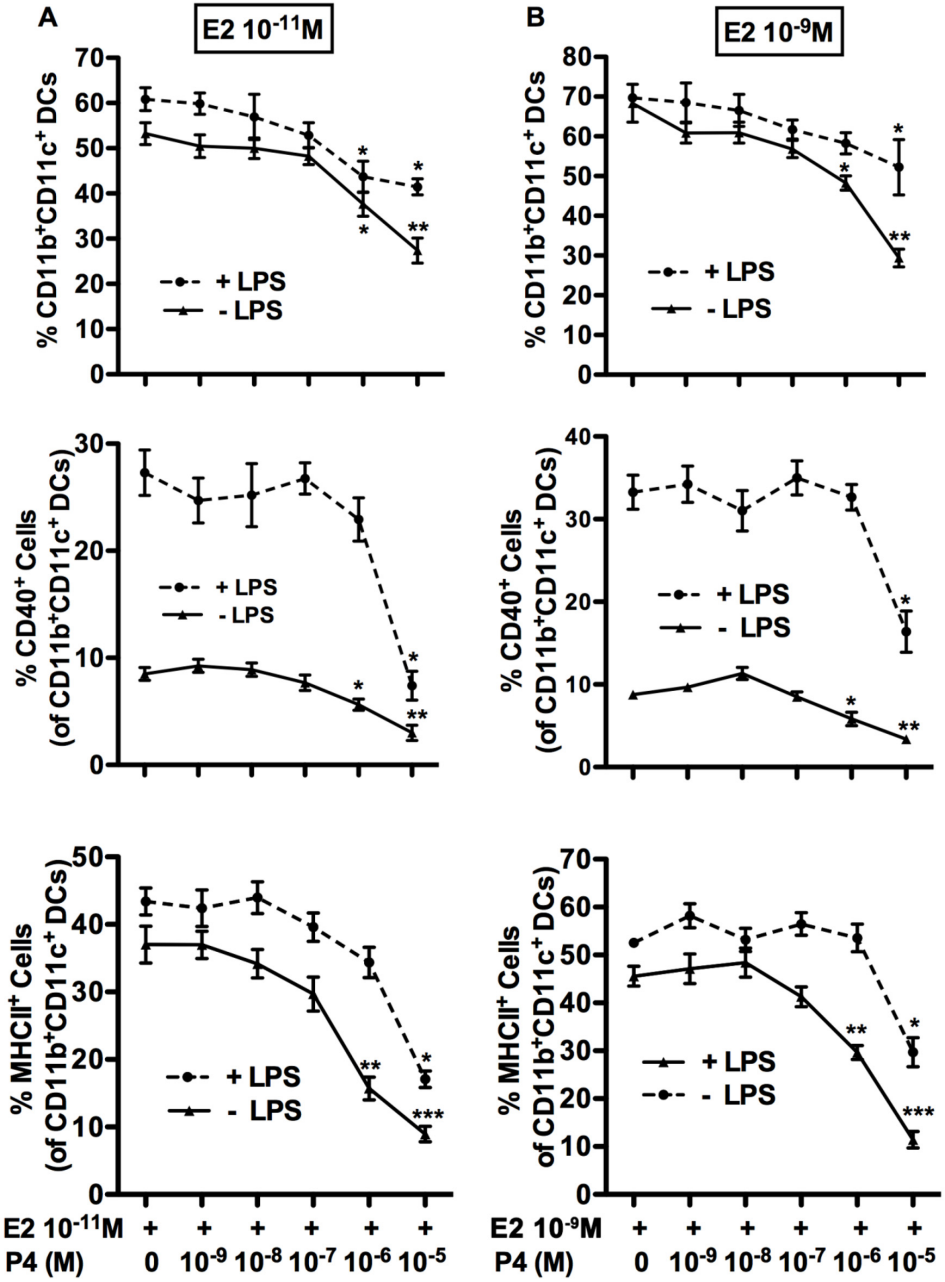
High doses of P4 reversed effects of E2 in presence or absence of LPS treatment. BM cells isolated from OVX mice were cultured with GM-CSF in the presence of E2 at a low (10^-11^M) (A) or high (10^-9^M) (B) concentration with varying doses of P4 (10^-9^M to 10^-5^M), with (dashed) or without (solid) LPS. At the end of culture, cells were harvested and stained with Abs to detect CD11b, CD11c, CD40 and MHCII. Data are percentages of CD11b^+^CD11c^+^ DCs (top panels); percentages of CD40^+^/CD11b^+^CD11c^+^DCs (middle panels); and percentages of MHCII^+^/CD11b^+^CD11c^+^ DCs (bottom panels). Data shown are Mean ± SD of 6 independent experiments. Statistical significance is indicated by *p<0.05; **p<0.01;*** p<0.001 compared with E2 only treated controls.

To better understand the role of the two sex hormones on DC differentiation and function, we investigated the combinatorial effects of E2 and P4. To this end, we added varying doses of P4 (from 10^-9^M to 10^-5^M) to either 10^-11^M (physiological) or 10^-9^M (high physiological, corresponding to pregnancy) of E2-treated BM cells on the first day of culture, in the presence or absence of LPS (Fig 4). The high doses of P4 corresponding to pregnancy level (10^-6^M) and supraphysiological level (10^-5^M) significantly decreased the percentage of CD11b^+^CD11c^+^ DCs and inhibited the percentage of CD11b^+^CD11c^+^ DCs expressing CD40 and MHCII, treated either with 10^-11^M (physiological) or 10^-9^M (pregnancy level) of E2. Interestingly, in the presence of LPS, supraphysiological dosages of P4 (10^-5^M) were required to reduce the frequency of mature DC expressing CD40 and MHCII, whereas 10^−6^ M of P4, corresponding to pregnancy levels, was sufficient to reduce the frequency of CD11b^+^CD11c^+^ cells. Thus, P4 was less effective in the presence of LPS at reversing E2-mediated differentiation of BMDC.

### Effects of E2 and P4 on BMDC differentiation are mediated by binding with their respective receptors

**Fig 5:**
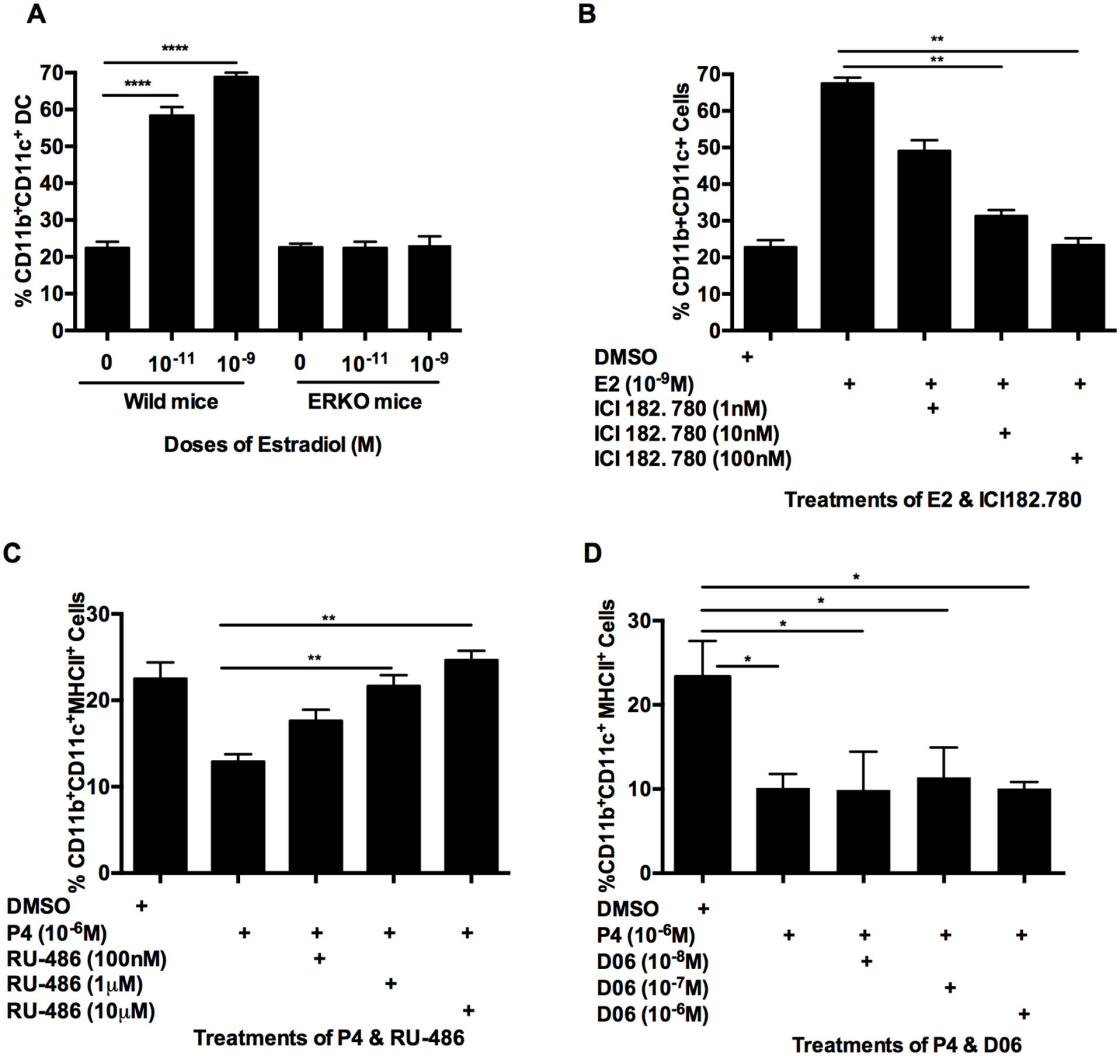
Effects of E2 and P4 on BM APC differentiation and maturation were mediated by their respective receptors. BM cells isolated from OVX mice (B, C, D) or ER KO mice (A) were cultured with GM-CSF in the presence of 10^-11^M (A) or 10^-9^M E2 (A, B) or (10^-6^M) P4 (C, D). Varying concentrations of E2 inhibitor ICI 182 780 (B), P4 inhibitor RU-486 (C) or Glucocorticoid receptor AL082D06 (D) were used in E2- or P4-treated BM cultures, beginning at the first day of culture. At the end of culture, cells were collected and stained with antibodies against CD11b and CD11c (A, B) and plus MHC II (C, D). Percentage of CD11c+ cells are shown in (A, B) for E2 cultured cells and P4 cultured cells (C, D). Data show Mean ± SD of triplicate determination each time of 3 independent experiments. Statistical significance is indicated by *p<0.05,** p<0.01, ***p<0.001 compared with untreated control.

To investigate if the effects of E2 or P4 are mediated through their respective receptors, we used estrogen receptor alpha knockout mice (ERKO) and receptor antagonists. As shown in Fig 5A, BM cells from ERKO mice did not respond to E2, showing no increase in frequency of CD11b^+^CD11c^+^ DC compared to BM cells from wild type mice. The specific antagonist for ER, ICI 182 780, was used to test if E2 increased BMDC differentiation by binding to its receptor. As shown in Fig 5B, as the amount of ICI 182 780 increased from 1 and 100 nM, the percentage of CD11b^+^CD11c^+^ DC decreased from over 60% to a background of ~20% (same level as BM cells without E2). The effect of progesterone was investigated using P4 receptor partial antagonist, RU-486. Because 10^-6^M of P4 did not significantly influence BM cell differentiation on its own (Fig 2), we used MHCII expression by mature BM APCs as a readout of P4 activity. As shown in the Fig 5C, as low as 1μM of RU-486 could reverse the inhibition of MHCII expression on P4-treated BM cells (p<0.01). Since the effect of P4 on MHCII expression was observed at higher concentrations, we decided to rule out that the effect could be due to P4 binding to glucocorticoid receptor (GR). 10^-6^M P4 was added to BM APCs in the presence of a GR-specific inhibitor (AL082D06), that has no known cross reactivity to any other steroid receptor [28]. In agreement with previous results, the percentage of BM APCs expressing MHCII was significantly decreased in P4 cultures, compared to hormone-naïve cultures (Fig 5D). Addition of different concentrations of D06 had no effect on percentage of BM cells expressing MHCII in presence of P4 (Fig 5D). These results demonstrated that the effects of E2 and P4 were specific to the hormones and mediated by binding with their respective receptors.

### Contrasting effects of E2 and P4 on antigen uptake of BMDC, pregnancy level concentrations of P4 inhibit E2 effects

**Fig 6:**
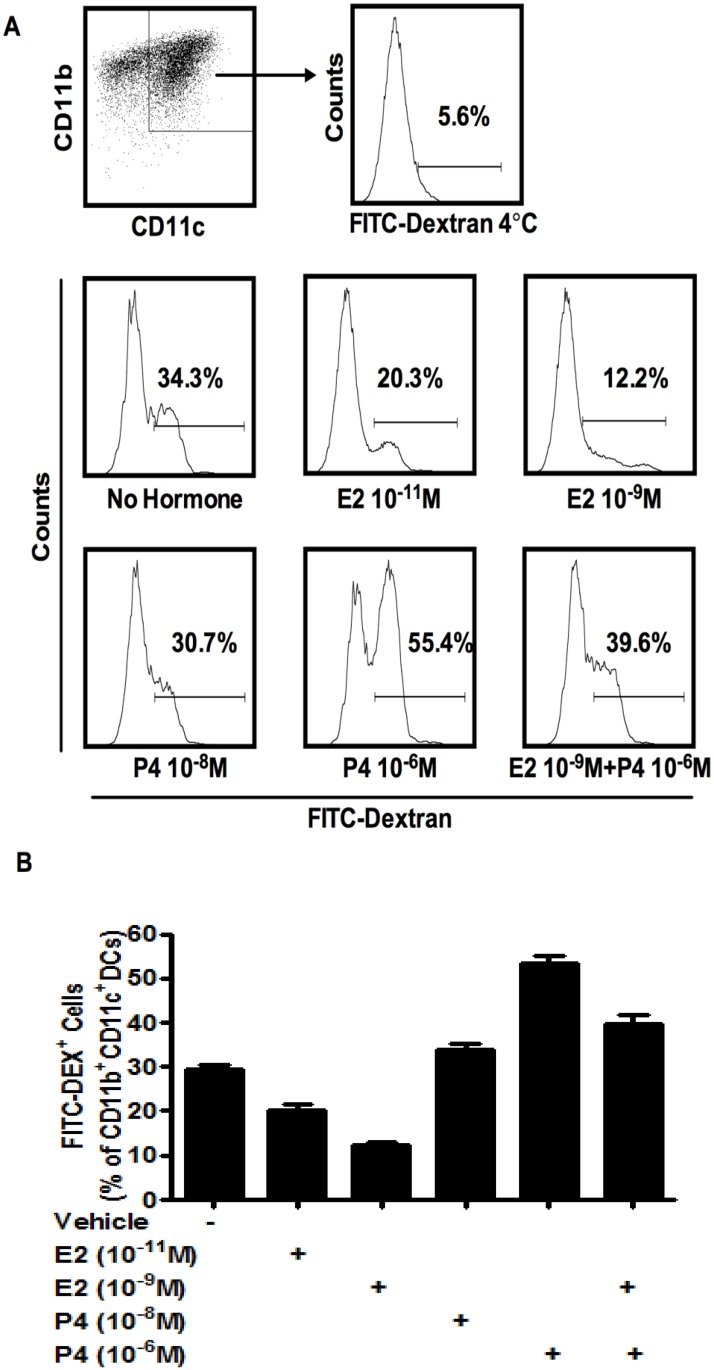
Effects of E2 and P4 on the uptake of FITC-Dextran by BMDC. BM cells isolated from OVX mice were cultured with GM-CSF in the presence of E2 (10^-11^M and 10^-9^M) or P4 (10^-8^M and 10^-6^M) or 10^-9^M E2 + 10^-6^M P4 and FITC-Dextran (200μg/ml) was added to the culture on day 5 for further 2 hrs at 37°C. Negative control was FITC-Dextran added to BMDCs and incubated at 4°C for 2 hrs. Cells were collected and stained with antibodies for CD11b and CD11c. Percentage of FITC-Dextran-positive CD11b^+^CD11c^+^ DCs were obtained by gating on CD11b^+^CD11c^+^ DCs. Representative flow cytometry data are shown in (A) and corresponding data graph are shown in (B). Data shown are Mean ± SD from 4 independent experiments. Statistical significance is indicated by *p<0.05; ** p<0.01; ***p<0.001 compared with untreated control or the comparison of combination treatment with single hormone treatment.

High antigen uptake ability is a characteristic of DCs prior to their activation and maturation [2]. Since our results indicated that BMDC cultures treated with E2 contained higher frequency of DCs expressing MHCII and CD40, markers expressed on mature DCs, whereas treatment with high physiological concentrations of P4 resulted in lower frequency of DCs expressing MHCII and CD40, we next investigated the effects of E2 and P4 on antigen uptake by DCs. BM cells cultured for five-days and treated with varying doses of E2 and P4, or their combination at pregnancy level concentrations, were incubated with FITC-Dextran, a typical model antigen that is endocytosed by immature DCs via the mannose receptor, which is expressed at high levels on immature DC [29]. As shown in Fig 6A, in the absence of any sex hormones, 34.3% of BMDC showed FITC-Dextran uptake. With the addition of physiological dose (10^-11^M) and pregnancy level (10^−9^ M) of E2, the percentage of FITC-Dextran labeled cells decreased to 20.3% and 12.2% respectively (Fig 6A), consistent with differentiation of a larger fraction of BMDC expressing CD40 and MHC II under these conditions (Fig 3). In contrast, treatment with pregnancy concentrations (10^-6^M) of P4 (Fig 6A) significantly increased the percentage of BMDC that labeled with FITC-Dextran, indicating decreased differentiation and/or presence of immature DCs in P4 treated cultures. Interestingly, when treated with a combination of pregnancy levels of both hormones (10^-9^M of E2 with 10^-6^M of P4), the percentage of BMDC labeled with FITC^-^Dextran was increased compared to 10^-9^M E2 alone, indicating that the effect of E2 was moderated by P4, when both hormones are present in this combination. These results were consistent with the opposing effects of E2 and P4 (Fig 4), with P4 effect dominating in cultures where BMDCs were treated with combination of both hormones.

### Differential effect of E2 and P4 on cytokine secretion from BMDC

**Fig 7:**
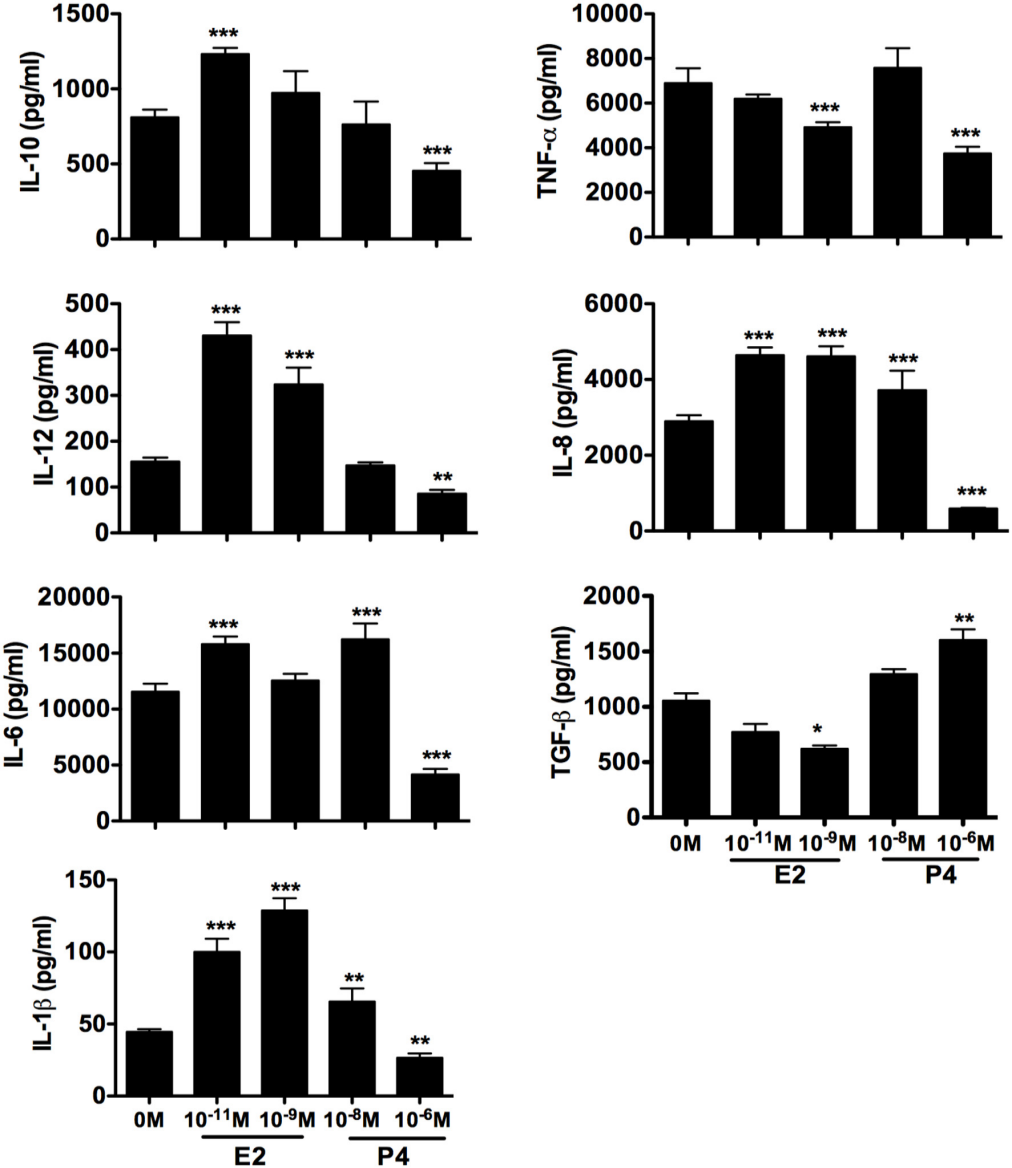
E2 and P4 induced different cytokine secretion profile from BMDCs. BM cells isolated from OVX mice were cultured with GM-CSF in the presence of varying doses of E2 or P4. On day 6, 5ng/ml LPS was added to the culture for further 24h culture and supernatants were collected and cytokine concentrations were determined (IL-12, IL-10, IL-8, IL-10, TNF-α, IFN-γ and IL-1β) by multi-analyte cytokine and chemokine assays. TGF-β in the supernatants was determined by ELISA. The values (pg/ml, mean ±SD) were normalized to the percentage of CD11b^+^ CD11c^+^ DCs in each treatment. Data represent triplicate samples from 2 independent experiments. Statistical significance is indicated by *p<0.05; ** p<0.01; ***p<0.001 compared with untreated control.

To determine the profile of cytokine secretion from BMDC treated with sex hormones, we collected supernatants from LPS activated BMDC treated with E2, P4, or combination of E2 and P4. Cytokines were chosen to represent those known to be secreted by DCs and play an important role in their functions: IL-1β, IL-6, IL-8, IL-10, IL-12, TNF-α, and TGF-β. We normalized the cytokine levels to the percentage of CD11b^+^CD11c^+^ DCs in each treatment culture, in order to better correlate BMDC with cytokine profile. Low physiological concentration of E2 (10^−11^ M) was effective at increasing IL-12, IL10, IL-8, IL-6 and IL-1β secretion from BMDC cultures, whereas high physiological concentrations of P4, corresponding to pregnancy levels (10^−6^ M), decreased secretion of all of these cytokines (Fig 7). The only cytokine where the effect of hormones was reversed was TGF-β; E2 was inhibitory for production of TGF-β such that pregnancy levels of 10^-9^M E2 actually inhibited TGF-β below the level of cultures containing no sex hormones. Cultures treated with high concentration of P4, equivalent of pregnancy levels (10^-6^M), showed the highest amount of TGF-β compared to all other culture conditions. TNF-α regulation was also unique, such that low concentrations of both E2 and P4 did not alter TNF-α secretion, but higher concentrations of both hormones suppressed TNF-α. The enhancing effect of E2 on IL-10, IL-12, IL-8 and IL-6 did not increase beyond that seen at low physiological dose (10^−11^ M), even with 100-fold increase in E2 concentrations.

**Fig 8:**
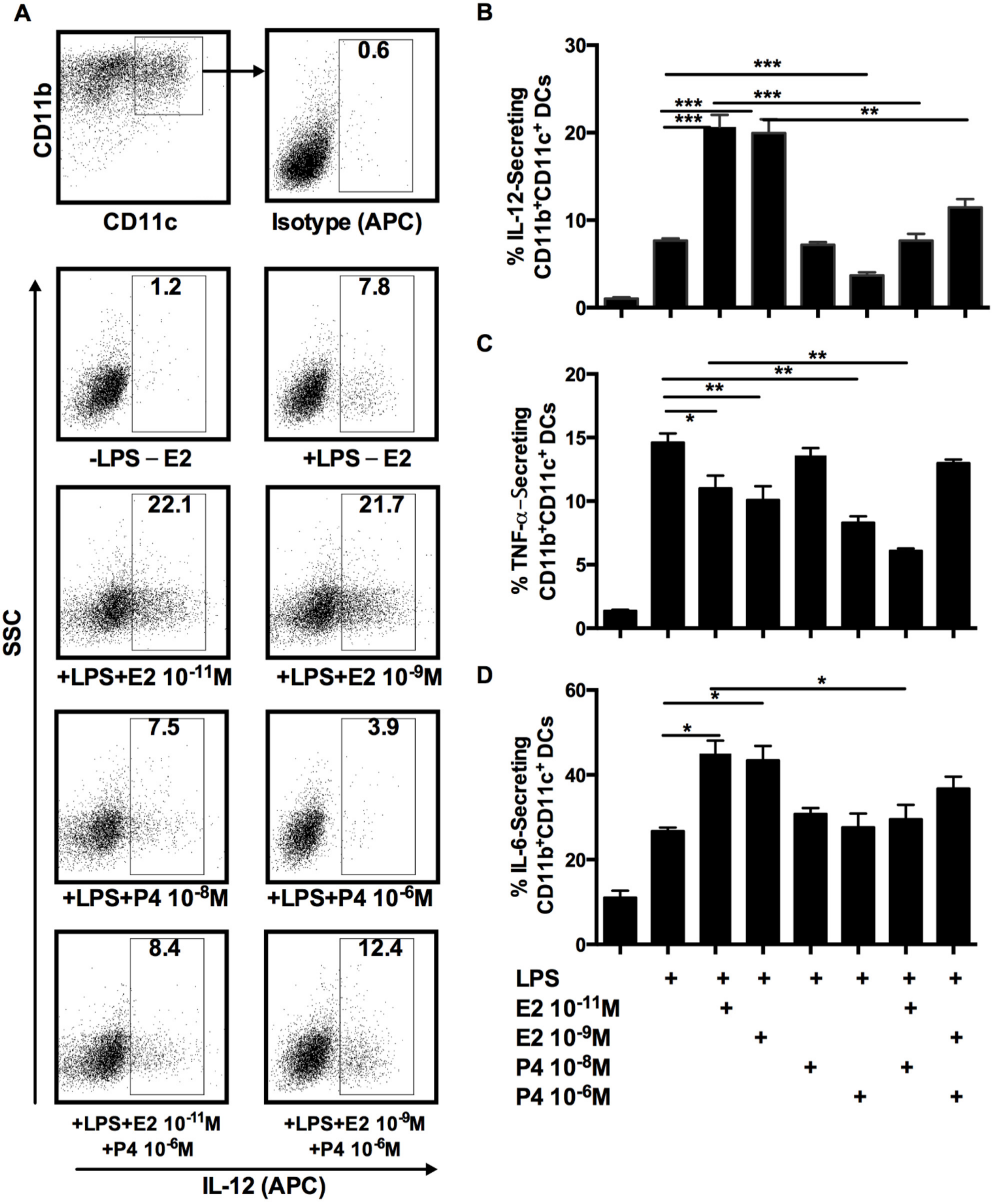
High dose of P4 inhibited the cytokine secretion from E2-treated BMDCs. BM cells isolated from OVX mice were cultured with GM-CSF in the presence of varying doses of E2 or P4 or combination of both. On day 6, 5ng/ml LPS was added to the culture for 24hrs. Culture treated with LPS alone (+LPS-E2) was negative control and untreated culture (-LPS -E2) was background. The protein transport inhibitor (BD Golgi Plug) was added for the last 6 hrs of culture. Cells were stained for intracellular IL-12, TNF- α and IL-6 with respective APC-conjugated antibodies. The top panel in A is gating scheme and isotype control. The rest of the panels in A are the percentage of IL-12-secreting CD11b^+^CD11c^+^ DCs by intracellular staining under experimental conditions. Percentage of CD11b+CD11c+ DCs positive for IL-12, TNF-α and IL-6 are showed in B, C and D, respectively. Data represent samples in triplicate from 2 independent determinations. *p<0.05; ** p<0.01; ***p<0.001 compared with non-hormone treated control or the comparison of combination treatment with single hormone treatment.

The cytokine analysis of culture supernatants reflected overall cytokine secretion by all the cell types present in the cultures. To define more accurately that the source of cytokine secretion was dendritic cells, we performed intracellular staining for cytokines IL-12, TNF-α and IL-6 on CD11c^+^CD11b^+^ BMDC. Representative dot plot data and corresponding graphs are shown (Fig 8A–8D). E2 treatment significantly increased frequency of DCs producing IL-12, but high dose of P4 decreased the frequency of IL-12 producing DCs. This clearly paralleled the results found in supernatant measures of IL-12 (Fig 7).

The graphic data of the frequencies of CD11c^+^CD11b^+^ BMDC secreting IL-6, and TNF-α showed a pattern generally similar to IL-12 (Fig 8C). Both high dose (10^−9^) and low dose (10^-11^M) of E2 increased BMDCs secreting IL-6, but only high dose of P4 (10^-6^M) decreased percentage of cells secreting IL-6 in the presence of low dose E2. For TNF-α, both low dose and high dose of E2 significantly decreased the frequency of cells secreting TNF-α (Fig 8D). In contrast, only high dose of P4 decreased TNF-α secreting cells on its own and in combination with low dose of E2.

## Discussion

Immune cells within the body are in constant contact with circulating hormones. While both female sex hormones, E2 and P4 co-occur throughout the reproductive years in a woman’s body, they have fluctuating concentration with E2 or P4 dominating at certain times of the reproductive cycle [21]. In addition, during pregnancy there is a prolonged period of P4 dominance at high concentrations [22]. Previous publications have illustrated the effects of either E2 or P4 on dendritic cells [30] [11]. All the studies so far have examined how E2 or P4 act on their own, and typically used one or two concentrations in the approximate physiologic range [25] [24] [16]. However, the interaction between these two hormones is very complex since they are present together, in vivo. While E2 and P4 have known synergistic effects, they can also antagonize each other since E2 upregulates P4 receptors and P4 can regulate ER [31] [32] [33]. Therefore, it is important to examine the combinatorial effect of these two hormones. We have now provided a full titration study of their individual effects on mouse BM progenitors, along with effect of combination of the two hormones at physiological concentrations that allow maximal effects on myeloid DC differentiation. Our data show clearly that E2 increases the differentiation of BM derived myeloid DC in vitro, at low physiological concentrations seen during normal reproductive cycle. Serum levels of E2 fluctuate between 10^−11^ to 10^-10^M (20–60 pg/ml) during the reproductive cycle, while P4 levels vary between 10^−9^ to 10^−8^ M (2–20 ng/ml) [21]. During pregnancy, serum E2 levels peak around 10^-9^M and P4 around 10^-6^M [22] [23]. Interestingly in follicular fluid and placenta, sites for local production of hormones in women, P4 levels have been reported to be as high as 9.8X10^-6^ M [26,27]. E2 increased differentiation of immature myeloid DC (CD11b+,CD11c+) from whole BM cell cultures at concentrations as low as 10^-12^M, with maximal effect (2–3 fold more than no hormone control) at 10^-9^M, in the presence or absence of LPS. LPS is a known stimulator of DC maturation in vitro and in vivo. A high percentage of the DCs generated in the presence of low E2 concentrations and LPS became functional DC with high CD40 and MHC Class-II expression along with secretion of cytokines such as IL-12, IL-10, and IL-1β. The reverse observations were found for BM cultures treated with P4 +/- LPS, at ranges from low physiologic to supraphysiologic (10^−9^–10^−5^ M). Frequency of CD11c+CD11b+ cells, percentage of cells expressing CD40 and MHCII and secreting cytokines decreased with high dosage of P4. More importantly, when combined at concentrations equivalent to those found in pregnancy, P4 inhibited the effects of E2. Thus our data indicates clearly that P4 is a regulator of the positive effects of E2 on myeloid DC differentiation and functions of BM precursors. Based on our data, one would predict increased differentiation of myeloid DCs of mature phenotype during estrogen-dominant stages and decrease in DC differentiation in progesterone-dominant stages. In agreement with this prediction, previous work has indicated that myeloid DC are present in large numbers during estrus in the mouse [34]. Similarly, previous studies have described the presence of a large number of immature DCs in the uterus of pregnant mice; whether this is a direct outcome of dominant progesterone levels seen during pregnancy remains to be proven in vivo [35].In this study we focused primarily on the effects of estradiol (E2) on DCs. However, in vivo, especially during pregnancy, levels of other estrogens, including estriol (E3) and estrone (E1) also increase significantly. However, similar to normal menstrual cycle, E2 is present in the highest concentration (peak levels 24ng/ml at week 35) throughout pregnancy, while E3 is the lowest (peak levels 6ng/ml at week 35) [36]. More interestingly, a comparison of total plasma level to free level showed that while E2 exists almost entirely in the unconjugated (free) form, estriol is present mostly in the inactive conjugated form compared to its total levels in plasma [36]. Other studies have confirmed these results [37]. Therefore, E2 is clearly responsible for majority of the estrogenic effects in pregnancy, whereas E3 is unable to exert much effect because it is predominantly conjugated. In addition to low free levels, E3 has been shown, in vitro, to have 12% the affinity of E2 for cytosolic ER [38]. In vivo studies in rats showed that E3 is a short acting compound with a brief duration of action and weak uterotrophic activity compared to E2 [38]. For these reasons, we limited our examination of estrogenic effects on DCs to E2.

A large body of work has focused on understanding the development, differentiation and functions of DCs using the protocol of BMDC cultures grown in presence of GMCSF with or without other cytokines and growth factors [39]. The large numbers of CD11c+ cells that differentiated under these conditions were considered to be pure myeloid DC population. However, a recent study identified heterogeneous groups of cells among the CD11c+ MHCII+ BMDCs, comprised of conventional myeloid DCs and monocyte derived macrophages [39]. Furthermore, both cell types underwent maturation with LPS, but responded differentially and showed distinct functional properties [39]. The study used CD135 and CD115 to define the subpopulations corresponding to DCs and macrophages, respectively. Since our studies were done prior to this work, we did not include any markers to distinguish whether we had mixed DCs and macrophages in our BMDC cultures. Therefore our results showing hormone effects on DCs need to be cautiously interpreted since the functional changes seen following different hormone treatments could be due to differential responses of DCs and macrophages. Nevertheless, since macrophages are present in abundant numbers in the uterus during normal cycle and make up 25–30% of the leukocytes in the decidua during pregnancy [40], understanding the differential effects of hormones on DCs compared to macrophages will be important for future studies.

In conclusion, we have addressed an existing gap in knowledge regarding the combinatorial effect of E2 and P4 on DC differentiation and function, given that under most physiological conditions these two hormones are present together. Our results indicate that while E2 effects dominate over lower physiological doses of P4 (10^−9^–10^−8^ M) such as those found during the reproductive cycle, while higher concentrations of P4 found in pregnancy (10^-6^M) have the dominant effect on BMDCs, decreasing their differentiation and moderating the production of inflammatory cytokines. These studies will provide a basis for better understanding of the regulation of DC differentiation and function by sex hormones.
